# A Longstanding Case of Black Grain Mycetoma in a Somalian Male Patient: A Case Report

**DOI:** 10.1002/ccr3.70394

**Published:** 2025-04-03

**Authors:** Mohamed Adam Mahamud, Mohamed Daffalla Awadalla Gismalla, Shafie Abdulkadir Hassan, Alaa Tajeldeen Habeeb Abdallah, Claude Mambo Muvunyi, Ayman Ahmed, Emmanuel Edwar Siddig

**Affiliations:** ^1^ Department of Medical Laboratory Sciences, Faculty of Medicine and Health Sciences Jamhuriya University of Science and Technology Mogadishu Somalia; ^2^ Department of Surgery, Faculty of Medicine University of Gezira Medani Sudan; ^3^ Department of Surgery, Faculty of Medicine Al‐Baha University Al‐Baha Saudi Arabia; ^4^ Rufa'a Teaching Hospital Al Gazeira State Sudan; ^5^ Rwanda Biomedical Centre Kigali Rwanda; ^6^ Institute of Endemic Diseases University of Khartoum Khartoum Sudan; ^7^ Faculty of Medical Laboratory Sciences University of Khartoum Khartoum Sudan

**Keywords:** buttock, eumycetoma, fungal infection, mycetoma, Somalia

## Abstract

Mycetoma is a chronic granulomatous infection predominantly affecting young individuals, characterized by painless swelling and often misdiagnosed due to its resemblance to other conditions. This case report presents the first documented instance of eumycetoma located in the buttock of a 44‐year‐old male patient from Somalia, a region with limited literature on this disease. The patient exhibited a 10‐year history of indurated swelling, which progressed to the discharge of dark granules, leading to the diagnosis of black grain eumycetoma. Comprehensive diagnostic methods, including imaging and histopathological analysis, were used, confirming the fungal etiology. Surgical excision followed, alongside antifungal therapy, resulting in significant improvement. This case emphasizes the necessity for increased awareness and consideration of mycetoma in clinical practice, especially in atypical presentations and underreported geographical areas.

**Summary**

- Clinicians should maintain a high index of suspicion for mycetoma in patients presenting with tumor‐like swellings, particularly in atypical locations, and ensure thorough diagnostic evaluation to facilitate timely treatment and prevent progression of this rare neglected tropical disease.

## 1 Introduction

Mycetoma is a chronic granulomatous infectious disease that is acquired through the implantation of the causative agents, either bacteria or fungal type, into the subcutaneous tissue, which eventually leads to the development of painless swelling, with or without discharging sinuses [1, 2, 3, 4]. The disease mainly affects the younger population, with the hands and feet being the most affected sites [4, 5, 6]. However, the infection can affect other body regions, including the leg, knee, forearm, and shoulder, with the chest, abdominal, and gluteal regions being the least affected sites [7, 8, 9, 10]. Mycetoma can clinically mimic many infectious and noninfectious diseases, including cutaneous tuberculosis, cutaneous schistosomiasis, leishmaniasis, and noninfectious conditions such as benign lesions and cancer of the skin [11, 12, 13, 14, 15, 16, 17, 18, 19]. Diagnosis can be made by the combination of clinical presentation, imaging, and laboratory‐based techniques such as culture, histopathology, Fine needle aspiration cytology, and, recently, molecular‐based techniques [20, 21, 22, 23, 24, 25]. There is scarce literature regarding this infection, and there are very few published cases of mycetoma in Somalia in the medical literature [26, 27, 28]. This case illustrates one patient with eumycetoma of the buttock, an atypical location, who was treated in the Department of Surgery, Mogadishu's Turkey Recep Tayyip Erdogan Training and Research Hospital.

## 2 Case History

A 44‐year‐old male patient from Bahdo district, Galgaduud region, Galmudug state, Somalia, presented with a large gluteal swelling reaching the perineal region lateral to the anus, which had been present for 10 years. Initially, the lesion was indurated and devoid of any opening. However, after a year and a half of unsuccessful treatments at various hospitals, the lesion developed an opening and began to discharge fluids comprising blood, pus, and, notably, dark granules resembling black grains.

## 3 Methods

Despite temporary relief following dressing at one facility, the wound re‐accumulated discharge and continued to worsen, prompting the patient to seek specialized treatment at Mogadishu's Turkey Recep Tayyip Erdogan Training and Research Hospital. On examination, a large swelling measuring over 10 cm was observed in the left buttock region, characterized by the discharge of dark fluid, blood, pus, and black granules. Remarkably, the patient reported discomfort associated with the lesion but denied the presence of systemic symptoms such as fever or malaise at the time of presentation.

Comprehensive laboratory investigations were conducted to ascertain the underlying etiology of the lesion. The results included renal and liver function within the normal range. Viral screening for HIV, hepatitis B, and C was negative. C‐reactive protein: 56 mg/L (indicating inflammation), white blood cell count: 5.6 × 10^3^/μL (within normal limits), red blood cell count: 4.34 × 10^6^/μL (within normal limits), hemoglobin: 11.5 g/dL (mild anemia), platelet count: 312 × 10^3^/μL (within normal limits), mean corpuscular volume (MCV): 26.5 fL, and mean corpuscular hemoglobin concentration: 31.6 g/dL (within normal limits). A swab was collected from the lesion for microbiological analysis, which resulted in the isolation of *Escherichia coli* from the wound discharge.

**FIGURE 1:**
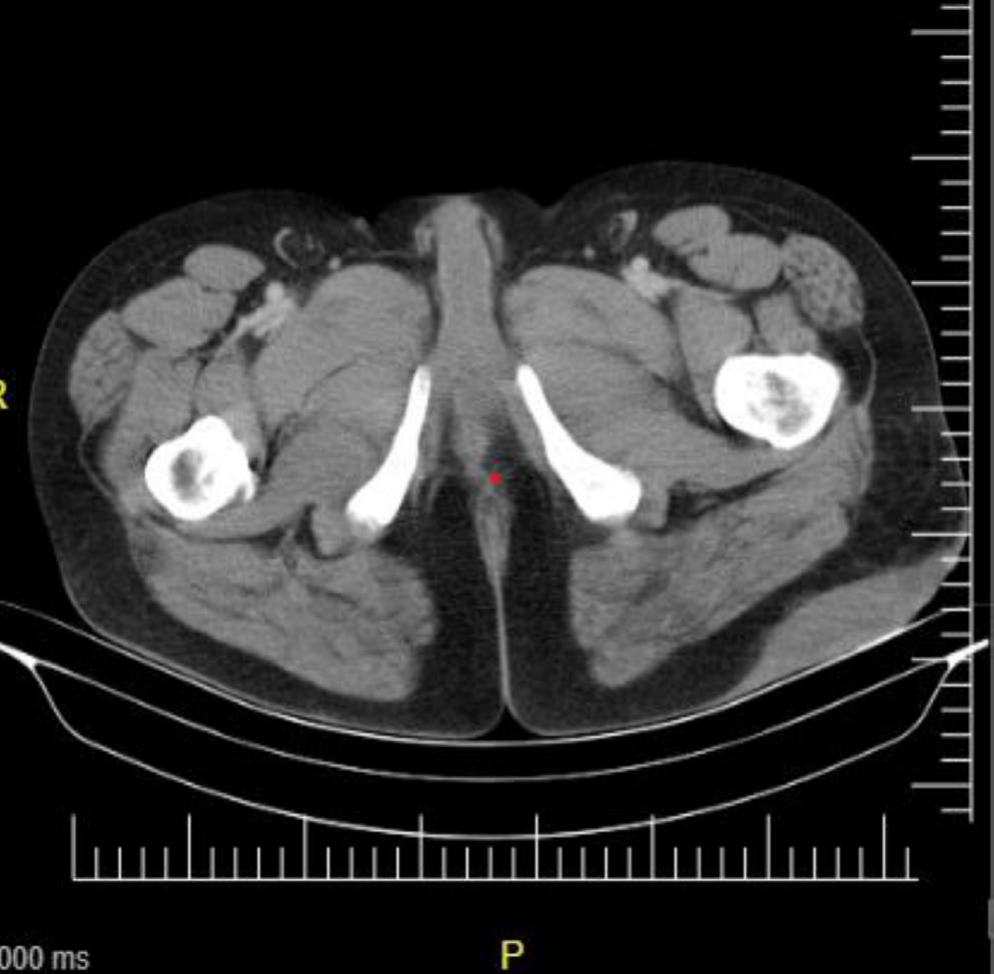
Computed Tomography (CT) scan of the patient revealing subcutaneous thickening in the left gluteal and proximal thigh regions, consistent with a soft tissue infection.

An ultrasound examination revealed hypoechoic collections in the left gluteal region, strongly suggesting the presence of an abscess. Subsequently, a computed tomography (CT) scan was performed, which confirmed subcutaneous thickening in the left gluteal and proximal thigh areas consistent with a soft tissue infection; the diagnosis was established as a gluteal tumefied mass with draining sinuses and tracts (Figure 1).

**FIGURE 2:**
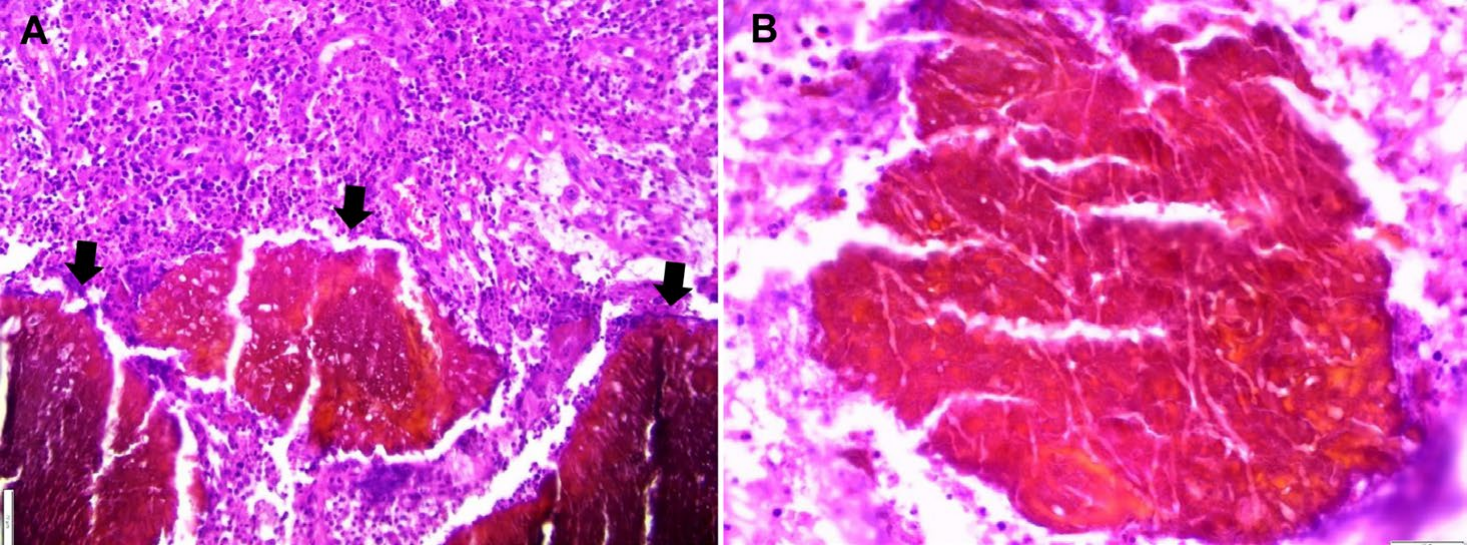
Histopathological images illustrating the microscopic characteristics of the lesion: (A) Several large dark brown grains (indicated by arrows) surrounded by inflammatory cells. (B) The grains demonstrate a filamentous fungal structure embedded within a cement‐like matrix (Hematoxylin and Eosin stain, magnifications: 10× and 40×).

Based on the clinical findings and investigations, we considered several differential diagnoses, including epidermal cyst, hidradenitis suppurativa, pilonidal cyst, cutaneous tuberculosis, cutaneous mycosis, leprosy, botryomycosis, and other metastatic cutaneous lesions. The patient was anesthetized by spinal anesthesia and put in a left lateral position. He underwent a wide surgical excision for a mycetoma lesion. The excised tissue was sent for histopathological analysis to exclude other potential lesions. Histological examination revealed features consistent with black grain eumycetoma, characterized by fungal elements and a mixed type II and III tissue reaction (Figure 2). These findings confirmed the diagnosis of mycetoma caused by black grains, indicating a fungal etiology.

## 4 Conclusion and Results

**FIGURE 3:**
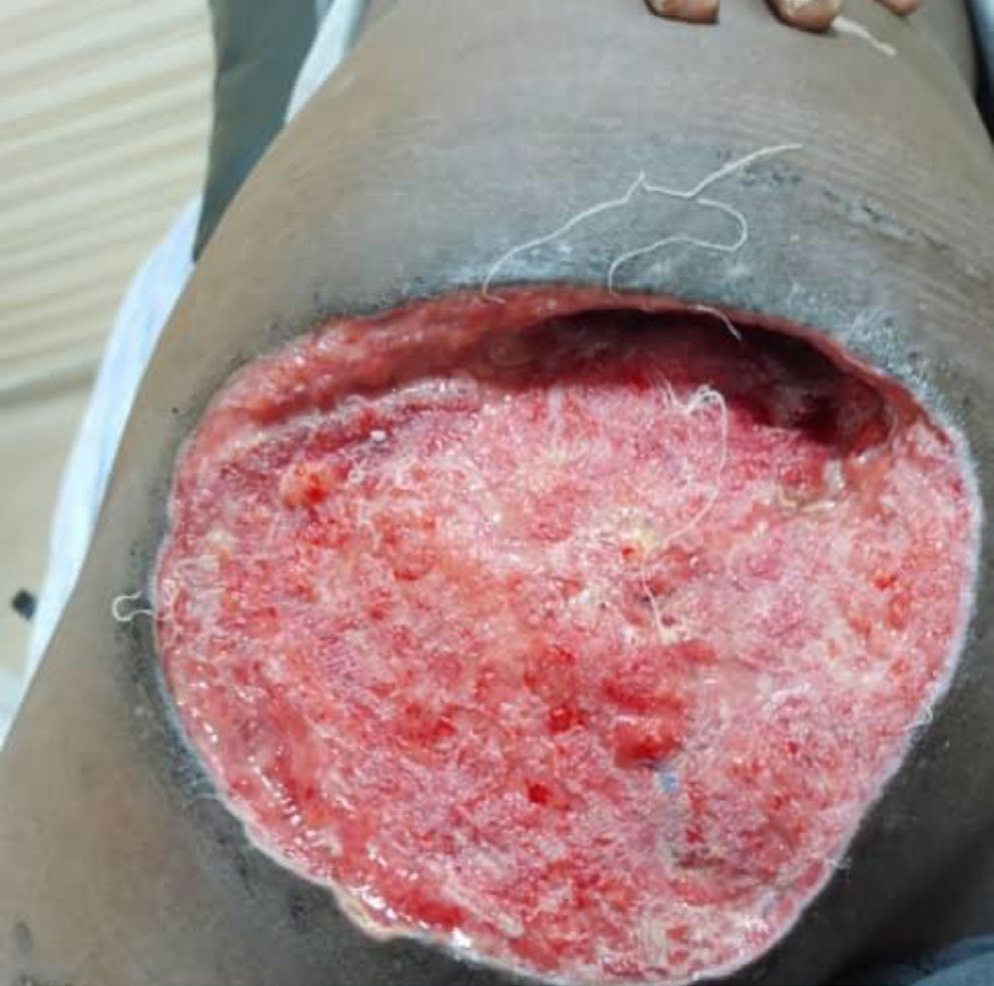
Post‐surgical photographic documentation of the lesion site after excision of the swelling, illustrating the condition of the area following intervention.

**FIGURE 4:**
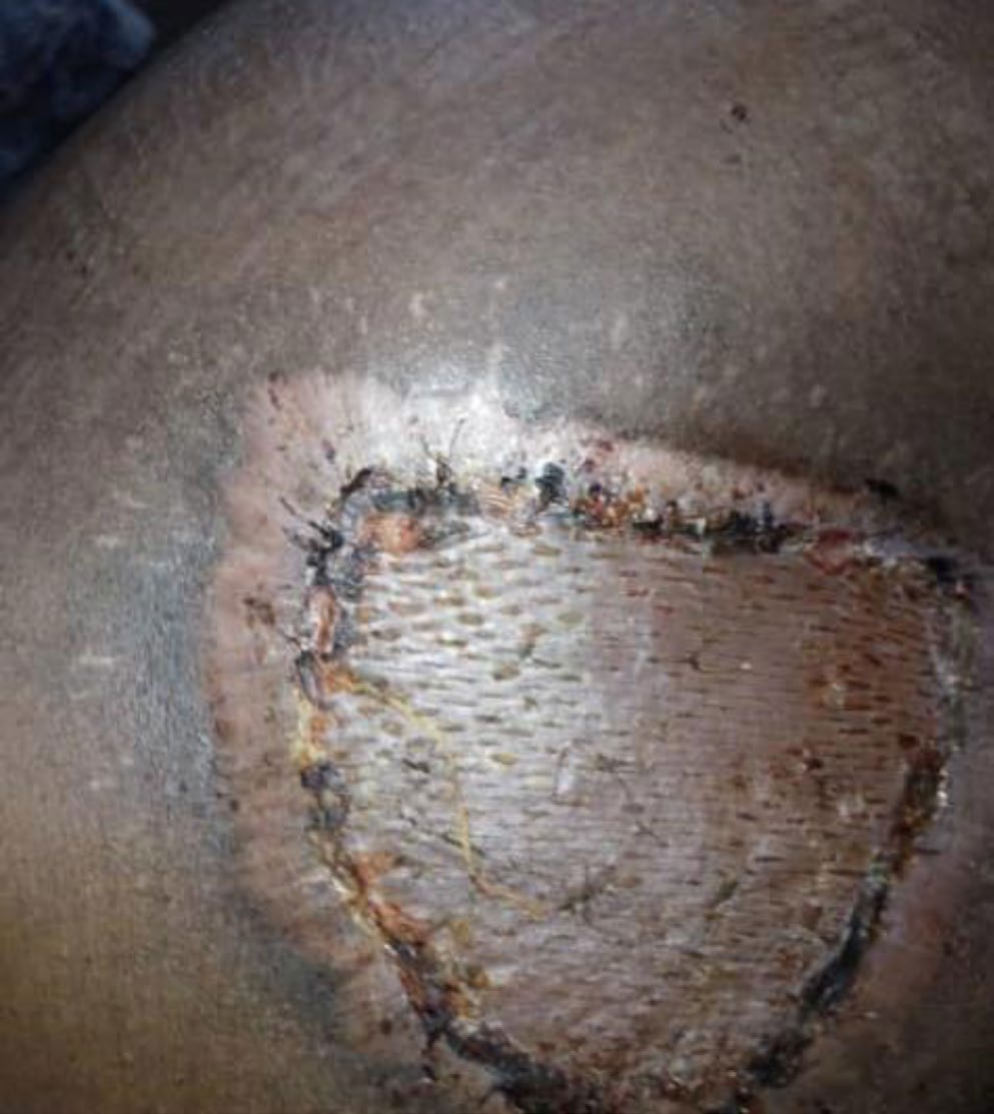
Illustrating the lesion after surgery and following the application of a skin graft.

The surgical procedure involved an elliptical incision with meticulous dissection to ensure the complete removal of the mass (Figure 3) followed by Skin graft. Postoperatively, a vacuum‐assisted closure system was implemented for effective wound management. This system utilizes a sterile gauze dressing placed directly onto the open wound, which is then covered by an adhesive film that seals both the dressing and the wound. A drainage tube, inserted through a hole in the adhesive film, connects to a portable vacuum pump. This pump creates continuous suction on the wound, promoting faster healing. Dressings are changed every four days. In terms of medication, the patient was prescribed paracetamol every six hours for two days, along with Amoxicillin‐clavulanate at a dosage of 875 mg every twelve hours. Additionally, itraconazole was prescribed at a dosage of 200 mg twice daily, specifically tailored for the treatment of eumycetoma. After three months of diligent management, the patient demonstrated significant improvement in both the wound condition and overall recovery (Figure 4).

## 5 Discussion

In this communication, we present the first reported case of eumycetoma involving the buttock, marking an unusual site for mycetoma in Somalia. This report is significant, as mycetoma is not well‐known in Somalia, with only a few cases documented in recent years [26, 27, 28]. It underscores the necessity for healthcare workers to be aware of mycetoma and to consider it in differential diagnoses for patients presenting with tumor‐like swellings, particularly in young men and in lower extremities. Although the disease can manifest in other locations in the body, visceral mycetoma has also been reported as well [29]. A high index of suspicion for mycetoma is essential, and appropriate sampling of lesions must include mycotic grains, which are crucial for diagnosis.

Clinically, mycetoma is characterized by a painless swelling during its initial phase, which can lead to diagnostic delays [1, 30, 31]. The swelling typically becomes painful only following a secondary bacterial infection or when the disease extends to the bone [30, 31]. Upon the inoculation of causative agents into the subcutaneous tissue, these organisms encapsulate themselves in a shield‐like pattern, forming grains that consist of the organisms and a cement‐like matrix [1, 3, 5]. This encapsulation serves as a protective mechanism against the host's immune system and antimicrobial agents [32]. The grains are considered pathogenomic and are essential for the histopathological differentiation of mycetoma from other subcutaneous infectious agents [1, 20, 33, 34]. Timely diagnosis is particularly crucial for infections located in critical areas such as the chest, abdomen, and buttocks.

Differential diagnoses for mycetoma include various conditions that may present similarly, such as abscesses, tumors (benign or malignant), and other fungal infections [11, 12, 13, 14, 15, 16, 17, 18, 19]. Therefore, an accurate and thorough clinical evaluation, alongside appropriate diagnostic tools, is vital for distinguishing mycetoma from these other entities.

Several diagnostic tools have been developed and validated in clinical settings, including culture, which is unfortunately time‐consuming, requiring 2–4 weeks for a definitive diagnosis [20, 21, 22, 23, 24, 25]. Additionally, it may be susceptible to contamination or fail to yield growth in certain cases, particularly in low‐income countries that may lack well‐established microbiological infrastructures. Histopathology remains a widely used diagnostic tool, as it is accessible in most settings, including resource‐limited areas [20, 21, 22, 23, 24, 25]. By conducting histopathological examinations, practitioners can readily differentiate mycetoma from non‐mycetoma lesions and distinguish between bacterial and fungal mycetoma.

In our case, the patient underwent two imaging studies: ultrasound and CT scan, both of which indicated the presence of infection. Ultrasound imaging proved particularly useful in medical centers with limited microbiological testing capabilities, as it effectively delineates the extent of the infection. However, it is essential to have trained radiologists who are familiar with the characteristic features of mycetoma to ensure accurate interpretation of the ultrasound findings. On the other hand, CT scans are generally more sensitive than ultrasound for assessing the extent of mycetoma within soft tissues. This sensitivity can provide crucial information for treatment planning [35, 36]. Therefore, the combination of these imaging modalities, along with the expertise of trained radiologists, enhances our ability to accurately evaluate and manage mycetoma cases.

The management of our case was based on surgical intervention, complemented by antifungal prophylaxis to minimize the risk of residual disease and recurrence. This is a well‐known and standard treatment modality [37]. This case highlights a black grain eumycetoma in the buttock region of a Somali patient. Although data concerning the burden of mycetoma in Somalia are scarce, the geographic proximity to Sudan warrants heightened awareness of this neglected tropical disease. Notably, mycetoma is not confined to the so‐called “mycetoma belt”; it is increasingly documented worldwide. Addressing this underreported disease requires improved awareness, education, and infrastructure to enable early diagnosis and effective treatment, ultimately preventing the progression to debilitating forms of this infectious disease. In conclusion, our findings advocate for a concerted effort to increase recognition and understanding of mycetoma within the healthcare community in Somalia and beyond, thereby ensuring timely intervention and potentially decreasing morbidity associated with this condition.

## Author Contributions

**Mohamed Adam Mahamud:** conceptualization, data curation, formal analysis, investigation, methodology, supervision, validation, visualization, writing – original draft, writing – review and editing. **Mohamed Daffalla Awadalla Gismalla:** conceptualization, data curation, formal analysis, investigation, methodology, supervision, validation, visualization, writing – original draft, writing – review and editing. **Shafie Abdulkadir Hassan:** conceptualization, data curation, formal analysis, investigation, methodology, supervision, validation, visualization, writing – original draft, writing – review and editing. **Alaa Tajeldeen Habeeb Abdallah:** conceptualization, data curation, formal analysis, investigation, methodology, supervision, validation, visualization, writing – original draft, writing – review and editing. **Claude Mambo Muvunyi:** conceptualization, data curation, formal analysis, investigation, methodology, supervision, validation, visualization, writing – original draft, writing – review and editing. **Ayman Ahmed:** conceptualization, data curation, formal analysis, investigation, methodology, supervision, validation, visualization, writing – original draft, writing – review and editing. **Emmanuel Edwar Siddig:** conceptualization, data curation, formal analysis, investigation, methodology, supervision, validation, visualization, writing – original draft, writing – review and editing.

## Disclosure

The authors have nothing to report.

## Consent

Written informed consent was obtained from the patient to publish this report in accordance with the journal's patient consent policy.

## Data Availability

The data that support the findings of this study are available from the corresponding author upon reasonable request.
